# Identification of *Lactobacillus* Strains Capable of Fermenting Fructo-Oligosaccharides and Inulin †

**DOI:** 10.3390/microorganisms9102020

**Published:** 2021-09-24

**Authors:** John A. Renye, Andre K. White, Arland T. Hotchkiss

**Affiliations:** Dairy & Functional Foods Research Unit, Agricultural Research Service, United States Department of Agriculture, Wyndmoor, PA 19038, USA; john.renye@usda.gov (J.A.R.J.); andre.white@usda.gov (A.K.W.)

**Keywords:** fermentation, prebiotic, probiotic, short-chain fatty acids, inulin, fructo-oligosaccharides, *Lactobacillus*

## Abstract

Novel probiotic strains that can ferment prebiotics are important for functional foods. The utilization of prebiotics is strain specific, so we screened 86 *Lactobacillus* strains and compared them to *Bifidobacterium breve* 2141 for the ability to grow and produce SCFA when 1% inulin or fructo-oligosaccharides (FOS) were provided as the carbon source in batch fermentations. When grown anaerobically at 32 °C, ten *Lactobacillus* strains grew on both prebiotic substrates (OD_600_ ≥ 1.2); while *Lactobacillus coryniformis* subsp. *torquens* B4390 grew only in the presence of inulin. When the growth temperature was increased to 37 °C to simulate the human body temperature, four of these strains were no longer able to grow on either prebiotic. Additionally, *L. casei* strains 4646 and B441, and *L. helveticus* strains B1842 and B1929 did not require anaerobic conditions for growth on both prebiotics. Short-chain fatty acid analysis was performed on cell-free supernatants. The concentration of lactic acid produced by the ten *Lactobacillus* strains in the presence of prebiotics ranged from 73–205 mM. *L. helveticus* B1929 produced the highest concentration of acetic acid ~19 mM, while *L. paraplantarum* B23115 and *L. paracasei* ssp. *paracasei* B4564 produced the highest concentrations of propionic (1.8–4.0 mM) and butyric (0.9 and 1.1 mM) acids from prebiotic fermentation. *L. mali* B4563, *L. paraplantarum* B23115 and *L. paracasei* ssp. *paracasei* B4564 were identified as butyrate producers for the first time. These strains hold potential as synbiotics with FOS or inulin in the development of functional foods, including infant formula.

## 1. Introduction

Lactic acid bacteria (LAB) are second only to yeasts as the most important group of microorganisms used worldwide by the food and feed industries. They serve as essential biocatalysts for production of fermented foods and their predominant metabolic end-product, lactic acid, has functioned as a natural food preservative for centuries. More recently, research has focused on the potential of LAB to improve human and animal health by serving as probiotics or producing natural bioactive food ingredients for the development of functional foods.

Well-characterized strains of lactobacilli and bifidobacteria are commercially available as human and animal probiotics, and additional strains, continue to be investigated for their potential to improve consumer health. Probiotics were reported to provide several health benefits to consuming hosts, including but not limited to: alleviation of lactose intolerance; lowering serum cholesterol; antioxidant, antihypertensive, anti-obesity and antidiuretic activities; immunomodulatory effects; and preventing the growth and colonization of microbial pathogens within the gastrointestinal tract [1]. Several of these health benefits were attributed to interactions between probiotics and the host epithelial or immune cells [2,3], or the indigenous microbiome [4,5].

Inulin and fructo-oligosaccharides (FOS) are non-digestible dietary fibers known to have prebiotic activity [6]. Inulin is a fructan with β-(2-1)-fructosyl chains and terminated by a glucosyl residue with an α-(1-2) linkage [7]. FOS can be enzymatically depolymerized inulin or produced from sucrose by transfructosylation [8]. Short-chain fatty acids produced by probiotic bacteria growing in the presence of prebiotics have several health benefits such as providing energy to colonic epithelial cells, inhibiting the growth of bacterial pathogens, and reducing secondary bile salt formation in the colon [9,10]. Synbiotics deliver both probiotics and prebiotics together, to maximize the beneficial effects of both entities [11]. Banning antibiotics as animal feed ingredients in Europe [12] led to increased interest in synbiotics for improving animal health [13], and several studies have reported on their potential health benefits within humans [14].

*Bifidobacteria* colonize the gut of breast-fed infants with *Bifidobacterium longum* subsp. *infantis*, *B. longum* subsp. *longum*, *B. breve*, *B. bifidum* and *B. pseudocatenulatum* most abundant, while formula-fed infants have a more diverse microbiome that also includes *B. adolescentis*, which is more common in adult guts [15,16,17]. *B. breve* and *B. bifidum* produce fucosidase and sialidase enzymes that are able to partially digest human milk oligosaccharides with a preference for lacto-N-tetraose [18], while *B. longum* subsp. *infantis* is the only strain capable of digesting all human milk oligosaccharide structures [19]. Human milk oligosaccharides may serve as prebiotics in the infant gut, and 2′ fucosyllactose is a commercial product used in infant formula. *B. longum* subsp. *infantis* is also commercialized as an infant probiotic supplement that improved diaper rash, colic, and sleep quality [20]. While the infant gut microbiome evolves continuously compared to the adult gut microbiome, infant probiotic supplementation may not have a long-term effect on colonization and health outcomes [21]. *Lactobacillus* present in breast milk also serves as a probiotic for the developing infant [22].

Several studies have shown that the ability to ferment prebiotic oligosaccharides varies for individual strains of *Lactobacillus* and *Bifidobacterium* species [23,24], thus LAB are continuously being screened for identification of novel strains which may function as probiotics individually or within a synbiotic. In this study, we screened 86 lactobacilli from an in-house culture collection to identify strains capable of fermenting inulin or FOS and characterized the SCFAs produced during fermentation. This work was performed to identify novel strains with the potential to serve as probiotic components in synbiotic applications.

## 2. Materials and Methods

### 2.1. Bacteria, Growth Media and Prebiotic Preparations

**Figure 1:**
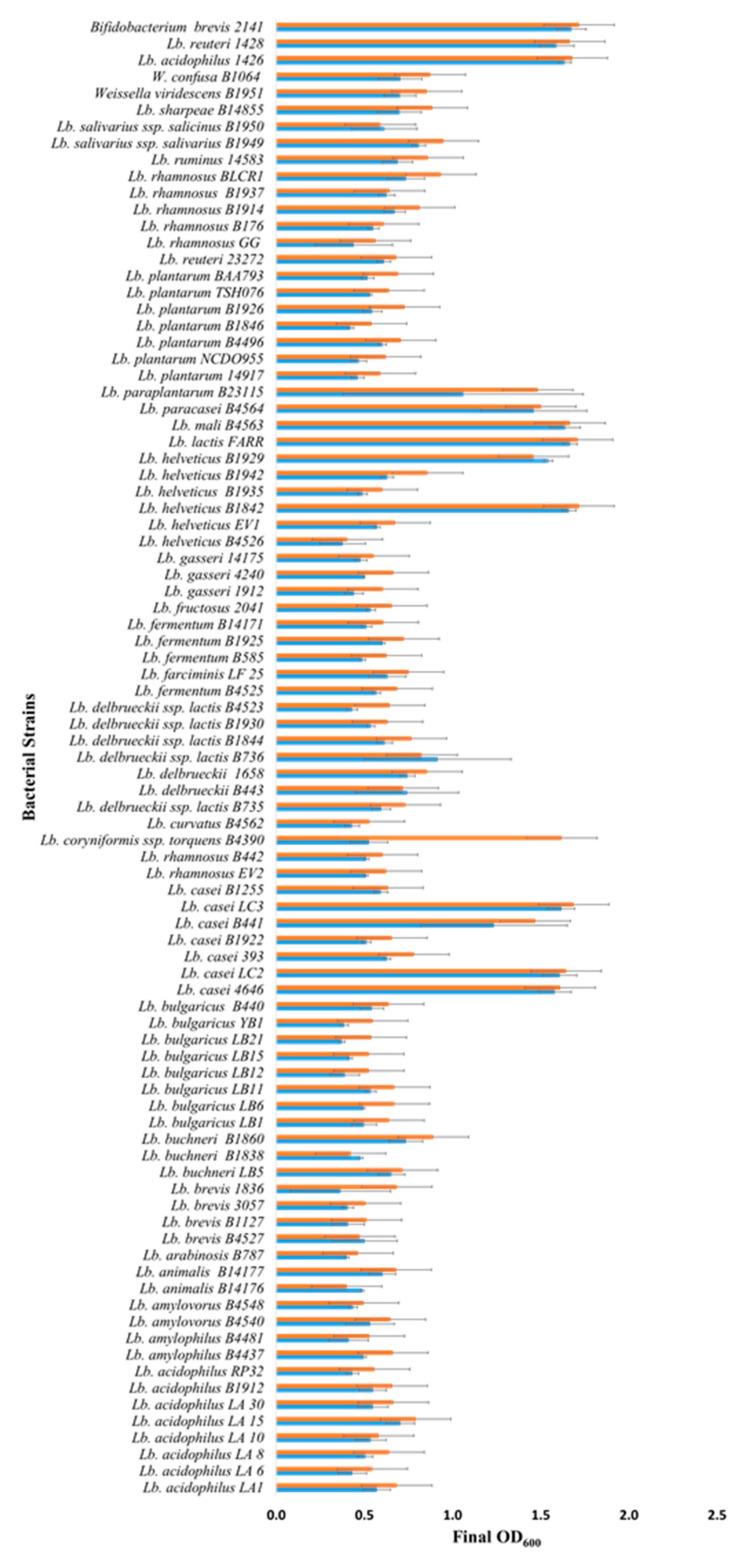
Growth of *Lactobacillus* and *Bifidobacterium* strains in mMRS broth (no glucose) supplemented with 1% FOS (blue bars) or inulin (orange bars) under anaerobic conditions. Bars represent the average optical density (600 nm) after incubation at 32 °C for 18–24 h (± standard deviation).

*Lactobacillus* strains and their sources are shown in Figure 1 and Table S1. The control strains *Lactobacillus acidophilus* 1426, *Lactobacillus reuteri* 1428 and *Bifidobacterium breve* 2141 were a gift from J. Luchansky (USDA, Wyndmoor, PA, USA). Bacteria were stored at −70 °C, and maintained in de Man, Rogosa, and Sharpe medium (MRS, Difco) at 32 °C; *B. breve* 2141 was routinely passaged under anaerobic conditions. A modified MRS medium (mMRS) (1.0% *w*/*v* proteose peptone No. 3; 1.0% *w*/*v* beef extract; 0.5% *w*/*v* yeast extract; 0.1% *w*/*v*; polysorbate 80; 0.2% *w*/*v* ammonium citrate; 0.5% *w*/*v* sodium acetate; 0.01% *w*/*v* magnesium sulfate; 0.005% *w*/*v* manganese sulfate; 0.2% *w*/*v* dipotassium phosphate) was prepared without glucose, to serve as the basal medium for prebiotic growth studies. Commercial prebiotics fructo-oligosaccharide (FOS) (Raftilose P95; Beneo, Parsippany, NJ, USA) and inulin (Raftilose Synergy 1; Beneo, Parsippany, NJ, USA) were obtained as powders and used to supplement basal mMRS at 1% *w*/*v*. The Raftilose P95 used was an oligo-fructose or an enzymatically digested inulin, while Raftilose Synergy 1 was a mixture of long-chain and short-chain inulin (Beneo, Parisippany, NY, USA). Solutions were filtered, sterilized (0.22 µm), and stored at 4 °C.

### 2.2. Bacterial Growth on Prebiotics

*Lactobacillus* (88) strains and *Bifidobacterium breve* 2141 were grown overnight in MRS broth at 32 °C. Cultures were washed twice in peptone water (0.1%), and then diluted 20-fold into 200 µL of mMRS, and mMRS containing 1% FOS or inulin. Bacteria were grown in the presence and absence of 10% Oxyrase (Oxyrase Inc., Mansfield, OH, USA), which was used to establish an anaerobic environment. Growth was monitored for 24 h at 32 or 37 °C in a Cytation 5 multimode plate reader (BioTek Instruments Inc., Winooski, VT, USA), with absorbance (600 nm) readings collected hourly. The data are the average optical density reading (OD_600nm_) from a minimum of two replicates (± standard deviation; SD).

### 2.3. Short-Chain Fatty Acid (SCFA) Analysis

*Lactobacillus* and *Bifidobacterium* strains were grown in mMRS containing 1% FOS or inulin (1 mL cultures) in the presence of 10% Oxyrase at 32 °C for 24 h. Cell free supernatants were collected by centrifugation at 13,000× *g* for 10 min and filtered (0.22 µm) prior to analysis by high performance liquid chromatography (HPLC) [11]. A 20 µL sample was injected and analyzed using an Aminex HPX-87H column with a micro-guard cation H guard cartridge (Bio-Rad, Hercules, CA, USA) and a RID-20A RI detector (Shimadzu Corp., Kyoto, Japan). The mobile phase was 5 mM sulfuric acid at a flow of 0.6 mL/min. Both column and RID were thermostated to 40 °C. Peaks for the known concentration of lactic, acetic, propionic, and butyric acids were determined and used as controls for calculating the concentrations of these SCFAs in mMRS prior and after fermentation with selected *Lactobacillus* strains and *Bifidobacterium breve* 2141. Results are the average of three runs of ±SD.

## 3. Results and Discussion

### 3.1. Lactobacillus Growth on FOS and Inulin

Batch culture fermentations using mMRS (no glucose) supplemented with 1% commercial FOS or inulin preparations identified 10 new *Lactobacillus* strains, out of the 86 screened, capable of growing on the prebiotics (Figure 1 and Table S1). These 10 strains: *Lactobacillus casei* strains 4646, LC2, LC3, and B441; *L. helveticus* strains B1842 and B1929; *L. lactis* FARR; *L. mali* B4563; *L. paracasei* B4564 and *L. paraplantarum* B23115 all reached a final optical density ≥1.0 after 18–24 h of growth under anaerobic conditions. These 10 *Lactobacillus* strains had the same growth as observed for *Bifidobacterium breve* 2141 in the presence of FOS and inulin (Figure 1). *B. breve* 2141 was previously reported to grow on MRS-FOS [24]. In addition, *L. coryniformis* subsp. *torquens* B4390 reached an optimal density of 1.6, but only in the presence of inulin. Several cultures reached an optical density between 0.5 and 0.8; however, they were also observed to grow (OD_600_ 0.3 and 0.5) in mMRS without prebiotic supplementation. This agreed with a previous study that reported lactobacilli grew on residual sugars present in a commercial FOS product or within basal MRS medium [24]. Lactobacilli which displayed intermediate growth (>0.6) in the presence of prebiotics and little or no growth on basal mMRS (OD_600_ ≤ 0.1), including *L. rhamnosus* strains B442 and B176; *L. delbreuckii* strains B735, B443, B1658 and B1844; *L. fermentum* B4524; *L. fructosus* 2041; *L. plantarum* B1926; *L. salivarius* strains B1949 and 1950; *L.* (*Weissella*) *viridescens* B1951 and *L.* (*Weissella*) *confuse* B1064 (Figure 1), warranted further investigation for their ability to metabolize FOS and inulin. Potentially, increasing the prebiotic concentration could improve bacterial growth, as previous studies have utilized FOS and inulin concentrations of 2% to support the growth of lactobacilli and bifidobacterial [24,25]. However, in the animal feed industry, supplements >1% are considered bulk ingredients and not functional feed ingredients [26].

*L. acidophilus* 1426 and *L. reuteri* 1428 were chosen as control strains for this study as FOS and inulin were previously shown to increase their survival within an alginate matrix designed for synbiotic applications [11]. In the current study, both strains were shown to grow on FOS and inulin resulting in optical densities >1.5 (Figure 1). These results suggest that their enhanced survival within an alginate matrix containing FOS or inulin [11] was maybe due to their ability to metabolize the prebiotics, rather than the result of a prebiotic-induced stress response which was reported to occur in *L. rhamnosus* [27]. Other studies have also reported the ability of both FOS and inulin to support the growth of *L. acidophilus* strains [24,28,29,30], thus it was surprising that the additional eight strains used in this study failed to grow in the presence of either prebiotic. Similarly, multiple strains of *L. bulgaricus* (8), *L. delbrueckii* (8), *L. plantarum* (7) and *L. rhamnosus* (9) were included in the current screen as all were reported to have grown on FOS or inulin [24,29,30,31]; however, none were capable of fermenting either prebiotic in this study (reaching an OD >1.0). This could be due to different chain lengths of inulin or FOS type (oligofructose or synthetic) used in these other studies. Our results further confirm that prebiotic utilization is strain dependent, and preliminary growth studies are required before attempting to utilize *Lactobacillus* strains for probiotic or synbiotic applications.

**Figure 2:**
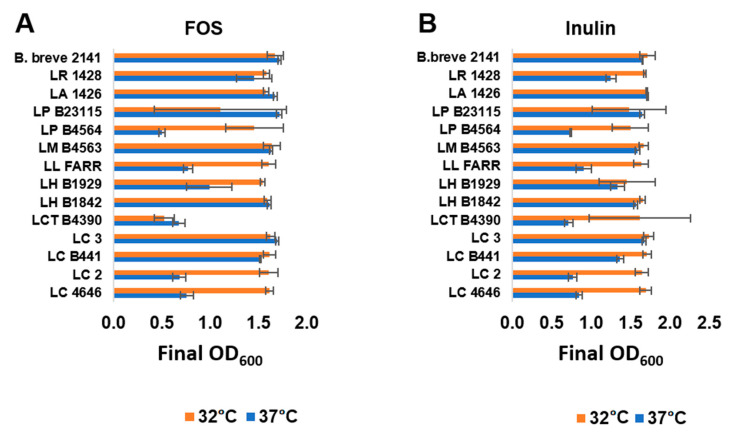
Growth of selected *Lactobacillus* strain at 32 °C (orange bars) or 37 °C (blue bars) in mMRS broth with (**A**) 1% FOS or (**B**) inulin under anerobic conditions. Bars represent the average optical density (600 nm) after 24 h of growth (± standard deviation).

The twelve FOS or inulin fermenter lactobacilli were further assessed for their ability to metabolize the prebiotics at 37 °C, which is representative of the temperature within the human GI tract. Fermentation of FOS and inulin at 32 and 37 °C was comparable for five (Figure 2A) and six (Figure 2B) strains grown on FOS and inulin respectively; as well as for all three control strains (*L. acidophilus* 1426, *L. reuteri* 1428 and *B. breve* 2141). *L. helveticus* B1929, which fermented both FOS and inulin comparable at 32 °C, did not reach as high an optical density at 37 °C when FOS was supplied as the carbohydrate source. *L. paracasei* B4564, *L. lactis* FARR and *L. casei* strains LC2 and 4646 fermented both prebiotics better at 32 °C, with cultures reaching an OD_600_ < 1.0 at 37 °C. *L. coryniformis* ssp. *torquens* B4390 fermented inulin better than FOS at 32 °C, while neither prebiotic was fermented well at 37 °C. Strains capable of metabolizing prebiotics at 37 °C would offer an advantage in the development of synbiotics by potentially enhancing probiotic growth within the colon and ultimately improving the targeted health benefit.

**Figure 3:**
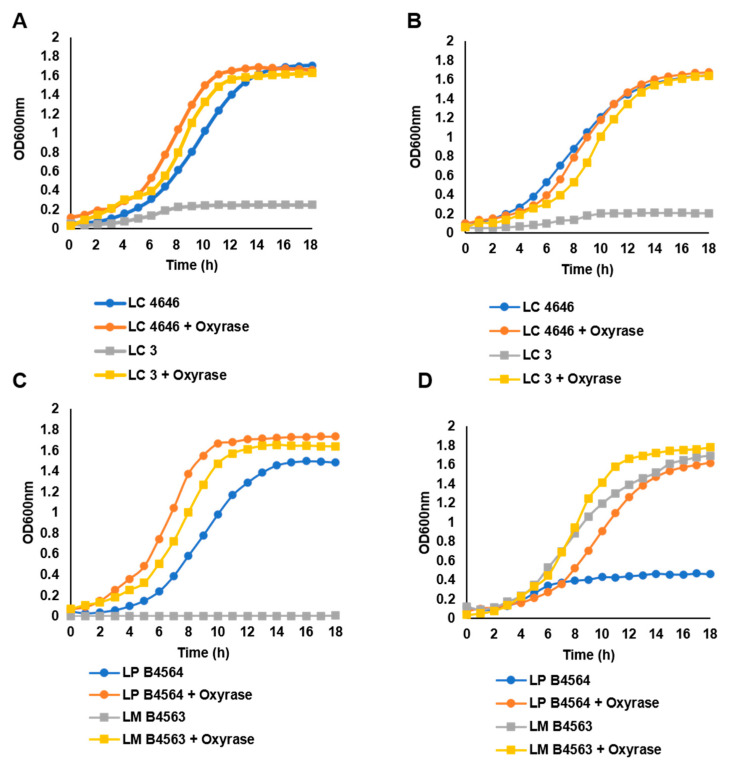
Growth of select *Lactobacillus* strains in mMRS broth containing (**A**,**C**) 1% FOS or (**B**,**D**) inulin at 32 °C in the presence (orange and yellow symbols) or absence (blue and gray symbols) of Oxyrase, which was used to generate anaerobic growth conditions. Strains shown: *L. casei* strains 4646 (LC4646); LC3; *L. paracasei* B4564 (LP B4564); and *L. mali* B4563 (LM B4563).

Growth on FOS and inulin was also assessed in the absence of Oxyrase, the enzyme used to remove dissolved oxygen from the cultures, to determine if the *Lactobacillus* strains required anaerobic conditions for prebiotic utilization. Five of the eleven strains shown to ferment FOS or inulin required anaerobic conditions for growth, including *L. coryniformis* ssp. *torquens* B4390, *L. lactis* FARR, *L. paraplantarum* B23115 and *L. casei* strains LC2 and LC3. In contrast to what was observed for *L. casei* strains LC2 and LC3, which reached a final OD_600_ < 0.5 when Oxyrase was omitted from the culture medium, the *L. casei* strain 4646 was not dependent on anaerobic conditions and reached an OD_600_ > 1.5 in the presence of oxygen (Figure 3A,B). The growth of *L. casei* B441, and *L. helveticus* strains 1842 and 1929 were comparable to *L. casei* 4646, in that anaerobic conditions were not required for optimal growth on either prebiotic. *L. mali* B4563 and *L. paracasei* B4564 were unique in that anaerobic conditions were essential for optimal growth on either inulin or FOS, respectively. *L. mali* B4563 was able to utilize inulin as a carbohydrate source in the presence of oxygen (Figure 3D); however, it only fermented FOS under anaerobic conditions (Figure 3C). For *L. paracasei* B4564, anaerobic conditions were essential for growth on inulin (Figure 3D), but not FOS (Figure 3C). A longer lag phase was observed for *L. paracasei* B4564 grown on FOS in the presence of oxygen, but a final OD_600_ > 1.4 was reached (Figure 3C). Additional molecular studies are required to understand why prebiotic utilization varied for *L. casei* strains, and *L. mali* B4563 and *L. paracasei* B4564. However, the ability of some strains to metabolize prebiotics in the presence of oxygen suggests they could be investigated for development of synbiotics aimed to improve human health at locations which are not strictly anaerobic, such as the oral cavity or skin. Previous studies have reported the potential for using probiotic lactobacilli and prebiotics to prevent infections and improve overall skin health [32], as well as to serve as supplements to the conventional treatment of dental caries [33].

### 3.2. Short-Chain Fatty Acid Production by Fermentation of FOS and Inulin

**Figure 4:**
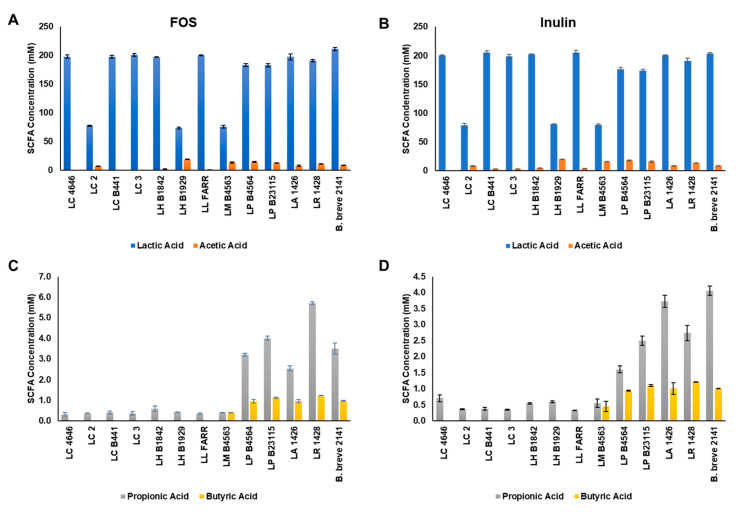
Short-chain fatty acid production by the ten *Lactobacillus* strains capable of fermenting both (**A**,**C**) FOS and (**B**,**D**) inulin. Bars represent the average concentration of each SCFA (nM): lactic (blue bars), acetic (orange bars), propionic acids (gray bars), and butyric acids (yellow bars) as the average of three replicates (±standard deviation).

*Lactobacillus* strains capable of growing on both FOS and inulin were investigated for production of lactic, acetic, propionic and butyric acids generated through fermentation. Lactic acid was the predominant end-product of fermentation for all *Lactobacillus* strains ranging from 74 to 205 mM (Figure 4A,B). *L. casei* stains 4646, B441, and LC3 all produced similar concentrations of lactic acid from the fermentation of FOS and inulin (>190 mM); however, strain LC3 produced significantly less lactic acid (78 mM) and the highest concentration of acetic acid (~8 mM) for the *L. casei* strains tested. Similar results were obtained for the two *L. helveticus* strains, with strain 1842 producing >190 mM lactic acid but only 2–5 mM acetic acid, and strain 1929 producing 73–81 mM lactic and >19 mM acetic acid. Of the remaining four strains tested, L. lactis FARR produced the highest concentration of lactic acid (~200 mM) and the lowest concentrations of acetic acid (0.3 mM with FOS; ~3 mM with inulin). *L. mali* B4563, *L. paracasei* B4564, and *L. paraplantarum* B23115 all produced 12–18 mM of acetic acid during FOS and inulin fermentation. A small amount of propionic acid was detected for all strains tested, but only *L. paracasei* B4564 and *L. paraplantarum* B23115, and the control strains *L. acidophilus* 1426, *L. reuteri* 1428 and B. breve 2141 produced concentrations >1 mM (Figure 4C,D). Butyric acid production was limited to *L. mali* B4563, *L. paracasei* B4564, *L. paraplantarum* B23115 and the control strains with concentrations ranging from an average of 0.4 to 1.1 mM. The highest concentration of butyric acid was detected after fermentation of FOS with the control strain *L. reuteri* 1428 (~1.3 mM).

The gut microbiome was shown to play an essential role in human metabolism and health [34]; and SCFAs produced from the fermentation of undigested dietary fibers were reported to regulate many of these functions [35]. The most commonly produced SCFAs include acetate, propionate, and butyrate, which accumulate within the cecum and proximal colon, and transported to peripheral tissues via the portal vein [36]. Accumulation of these SCFAs were reported to contribute to the maintenance of a healthy gut microbiota by inhibiting the growth and colonization of bacterial pathogens [37,38]; and regulating the production of mucin [39,40]. Butyrate was also reported to serve as an energy source for colonocytes; strengthen the immune system by increasing regulatory T-cell generation within colon; and prevent intestinal bowel disease and colorectal cancer through its anti-inflammatory activity [35]. When entering peripheral tissues, these SCFA were associated with lowering serum cholesterol, preventing obesity and reducing incidents of chronic obstructive pulmonary disease [41,42]. However, intestinal dysbiosis may complicate the use of prebiotics to generate beneficial SCFAs from the indigenous gut microbiome, thus synbiotics were explored for improving human health by delivering a probiotic in the presence of prebiotic fibers that it is capable of fermenting [43]. This study identified ten strains capable of fermenting commercial preparations of inulin and FOS, with nine of these strains warranting further investigation as potential candidates for synbiotic applications. The exception being *L. casei* 4646 which was reported to contribute to dental caries formation [44]. Several reports have described the probiotic potential for other strains of *L. casei* [45,46,47], *L. helveticus* [48], *L. paracasei* [49], *L. mali* [50,51] and *L. paraplantarum*; however, the literature remains limited on the potential for these *Lactobacillus* species to ferment prebiotic fibers for production of SCFAs. Previous studies have reported *L. casei* strains capable of fermenting FOS [23,24,52], and *L. paracasei* strains capable of fermenting FOS [53] or inulin [47,54]. In one study, organic acid production was monitored and *L. casei* strain ASCC 292 was shown to produce acetic, propionic, and butyric acids [52]. These results differed from what was observed in this study, as the four *L. casei* strains shown to ferment FOS and inulin did not produce butyric acid and produced significantly less acetic and propionic acids than reported earlier [28]. These differences may be due to the varying metabolic activities for each strain, but the Liong and Shah [52] study also determined the optimal culture conditions for production of these SCFAs. Additional research is required to define the conditions for the optimal production of SCFAs from the *Lactobacillus* strains used in our study. Another study demonstrated the prebiotic potential of native and commercial inulin for supporting the growth of *L. paraplantarum* strains; however, growth on FOS was not assessed [55].

## 4. Conclusions

To our knowledge, this is the first study to report on the identification of *L. helveticus*, *L. lactis*, *L. paraplantarum*, and *L. mali* strains capable of fermenting both FOS and inulin. While these results suggest the potential for using these strains for the development of synbiotics with FOS or inulin, more work is required to demonstrate their potential as probiotics. Future work will also focus on optimizing their production of SCFAs, specifically *L. mali* B4563, *L. paraplantarum* B23115, and *L. paracasei* B4564 which were shown to produce acetic, propionic, and butyric acids. Butyric acid production by these three strains was comparable to the concentrations produced by *Bifidobacterium breve* 2141 and *Lactobacillus paracasei* D3-5, which were used to develop a probiotic cocktail consisting of lactobacilli (5 strains) and enterococci (5 strains) to modulate the human microbiome by increasing SCFA concentrations [56]. Additionally, the ten strains capable of fermenting both prebiotics will be used in future studies to assess the prebiotic potential of novel oligosaccharides isolated from a variety of food sources. Future studies with these ten *Lactobacillus* strains in the presence of milk will assess their use in infant formula applications.

## Data Availability

Not applicable.

## Supplementary Materials

The following are available online at https://www.mdpi.com/article/10.3390/microorganisms9102020/s1, Table S1: Growth of *Lactobacillus* and *Bifidobacterium* species in mMRS broth supplemented with 1% FOS or inulin.
