# Supplementary material for: Identification of Lactobacillus Strains Capable of Fermenting Fructo-Oligosaccharides and Inulin
Source: Microorganisms. 2021 Sep 24;9(10):2020. doi: 10.3390/microorganisms9102020 (PMC8537702; doi:10.3390/microorganisms9102020)
Supplement: Supplementary file 1 [file microorganisms-09-02020-s001.zip › microorganisms-1380081-supplementary.pdf]

microorganisms

MDPI

Supplementary Materials:

Table S1: Growth of *Lactobacillus* and *Bifidobacterium* species in mMRS broth supplemented with 1% FOS or inulin.

Bacteria	Source[1]	P95 (FOS)		Synergy 1 (Inulin)	
		OD 600[2]	± SD	OD600[2]	± SD
L. acidophilus LA1	DFF	0.570	0.077	0.684	*0.002*
L. acidophilus LA 6	DFF	0.432	0.081	0.545	*0.195*
L. acidophilus LA 8	DFF	0.505	0.043	0.640	*0.001*
L. acidophilus LA 10	DFF	0.537	0.087	0.581	*0.142*
L. acidophilus LA 15	DFF	0.704	0.081	0.791	*0.050*
L. acidophilus LA 30	DFF	0.549	0.086	0.663	*0.001*
L. acidophilus B1912	NRRL	0.548	0.077	0.657	*0.028*
L. acidophilus RP32	DFF	0.432	0.036	0.557	*0.001*
L. amylophilus B4437	NRRL	0.496	0.013	0.661	*0.053*
L. amylophilus B4481	NRRL	0.410	0.113	0.527	*0.064*
L. amylovorus B4540	NRRL	0.532	0.138	0.647	*0.081*
L. amylovorus B4548	NRRL	0.436	0.024	0.495	*0.022*
L. animalis B14176	NRRL	0.487	0.010	0.399	*0.011*
L. animalis B14177	NRRL	0.602	0.076	0.679	*0.135*
L. arabinosis B787	NRRL	0.400	0.013	0.463	*0.040*
L. brevis B4527	NRRL	0.501	0.184	0.473	*0.042*
L. brevis B1127	NRRL	0.406	0.093	0.513	*0.065*
L. brevis 3057	DFF	0.406	0.033	0.506	*0.059*
L. brevis 1836	DFF	0.364	0.285	0.684	*0.111*
L. buchneri LB5	DFF	0.654	0.076	0.716	*0.101*
L. buchneri B1838	NRRL	0.477	0.014	0.422	*0.073*
L. buchneri B1860	NRRL	0.736	0.095	0.890	*0.114*
L. bulgaricus LB1	DFF	0.498	0.071	0.639	*0.008*
L. bulgaricus LB6	DFF	0.494	0.009	0.670	*0.051*
L. bulgaricus LB11	DFF	0.537	0.030	0.670	*0.080*
L. bulgaricus LB12	DFF	0.389	0.083	0.524	*0.107*
L. bulgaricus LB15	DFF	0.417	0.015	0.524	*0.057*
L. bulgaricus LB21	DFF	0.372	0.017	0.537	*0.028*
L. bulgaricus YB1	DFF	0.386	0.024	0.546	*0.006*
L. bulgaricus B440	NRRL	0.542	0.067	0.636	*0.070*
L. casei 4646	*ATCC*	1.579	0.093	1.609	*0.141*
L. casei LC2	DFF	1.608	0.097	1.644	*0.085*
L. casei 393	ATCC	0.630	0.019	0.780	*0.028*
L. casei B1922	NRRL	0.513	0.025	0.655	*0.064*
L. casei B441	NRRL	1.235	0.416	1.469	*0.357*
L. casei LC3	DFF	1.617	0.076	1.686	*0.062*

L. casei B1255	NRRL	0.593	0.039	0.634	*0.120*
L. rhamnosus EV2	DFF	0.509	0.011	0.624	*0.032*
L. rhamnosus B442	NRRL	0.513	0.013	0.603	*0.072*
L. coryniformis ssp. *torquens* B4390	NRRL	0.526	0.107	1.619	*0.640*
L. curvatus B4562	NRRL	0.430	0.041	0.527	*0.058*
L. delbrueckii ssp. *lactis* B735	NRRL	0.596	0.054	0.731	*0.193*
L. delbrueckii B443	NRRL	0.742	0.292	0.719	*0.063*
L. delbrueckii B1658	NRRL	0.745	0.042	0.855	*0.033*
L. delbrueckii ssp. *lactis* B736	NRRL	0.916	0.415	0.826	*0.053*
L. delbrueckii ssp. *lactis* B1844	NRRL	0.615	0.045	0.766	*0.049*
L. delbrueckii ssp. *lactis* B1930	NRRL	0.536	0.023	0.632	*0.064*
L. delbrueckii ssp. *lactis* B4523	NRRL	0.430	0.032	0.643	*0.095*
L. fermentum B4525	NRRL	0.569	0.022	0.686	*0.066*
L. farciminis LF 25 (B4566)	NRRL	0.630	0.106	0.749	*0.221*
L. fermentum B585	NRRL	0.490	0.016	0.625	*0.045*
L. fermentum B1925	NRRL	0.605	0.012	0.723	*0.170*
L. fermentum B14171	NRRL	0.513	0.029	0.607	*0.122*
L. fructosus 2041	DFF	0.536	0.026	0.656	*0.106*
L. gasseri 1912	DFF	0.441	0.054	0.605	*0.088*
L. gasseri 4240	DFF	0.498	0.004	0.663	*0.044*
L. gasseri 14175	DFF	0.479	0.035	0.553	*0.062*
L. helveticus B4526	NRRL	0.377	0.130	0.403	*0.065*
L. helveticus EV1	DFF	0.573	0.016	0.674	*0.078*
L. helveticus B1842	NRRL	1.661	0.040	1.716	*0.101*
L. helveticus B1935	NRRL	0.489	0.028	0.601	*0.054*
L. helveticus B1942	NRRL	0.630	0.037	0.858	*0.017*
L. helveticus B1929	NRRL	1.543	0.025	1.458	*0.354*
L. lactis FARR	OSU	1.666	0.041	1.708	*0.722*
L. mali B4563	NRRL	1.638	0.087	1.665	*0.059*
L. paracasei B4564	NRRL	1.460	0.300	1.500	*0.230*
L. paraplantarum B23115	NRRL	1.060	0.681	1.482	*0.470*
L. plantarum 14917	*ATCC*	0.461	0.036	0.590	*0.057*
L. plantarum NCDO955	DFF	0.468	0.045	0.622	*0.063*
L. plantarum B4496	NRRL	0.601	0.023	0.706	*0.075*
L. plantarum B1846	NRRL	0.422	0.017	0.540	*0.041*
L. plantarum B1926	NRRL	0.545	0.054	0.729	*0.091*
L. plantarum TSH076	NRRL	0.532	0.011	0.639	*0.061*
L. plantarum BAA793	ATCC	0.519	0.036	0.692	*0.005*
L. reuteri 23272	ATCC	0.611	0.039	0.680	*0.109*
L. rhamnosus GG	ATCC	0.440	0.219	0.562	*0.316*
L. rhamnosus B176	NRRL	0.551	0.031	0.608	*0.023*
L. rhamnosus B1914	*NRRL*	0.672	0.061	0.812	*0.033*
L. rhamnosus B1937	NRRL	0.626	0.048	0.642	*0.045*

L. rhamnosus BLCR1	DFF	0.735	0.107	0.934	*0.076*
L. ruminus 14583	DFF	0.689	0.086	0.860	*0.045*
L. salivarius ssp. *salivarius* B1949	NRRL	0.808	0.038	0.948	*0.207*
L. salivarius ssp. *salicinus* B1950	NRRL	0.613	0.185	0.590	*0.256*
L. sharpeae B14855	NRRL	0.699	0.123	0.884	*0.416*
L. (Weissella) viridescens B1951	NRRL	0.703	0.091	0.852	*0.024*
L. (Weissella) confusa B1064	NRRL	0.703	0.125	0.872	*0.059*
L. acidophilus 1426	Luchansky	1.635	0.036	1.678	*0.044*
L. reuteri 1428	Luchansky	1.590	0.098	1.664	*0.035*
Bifidobacterium breve 2141	ATCC 15698	1.674	0.082	1.716	*0.095*

[1]Source for bacterial cultures used in this study: DFF: Dairy and Functional Foods Research Unit, Eastern Regional Research Center, U.S. Department of Agriculture, Wyndmoor, PA; NRRL: Northern Regional Research Center, The National Center for Agriculture Utilization Research, U.S. Department of Agriculture, Peoria, IL; ATCC: American Type Culture Collection, Rockville, MD; OSU: Oregon State University, gift from W. E. Sandine; Luchansky: John Luchansky, Eastern Regional Research Center, U.S. Department of Agriculture, Wyndmoor, PA, ATCC: American Type Culture Collection

[2] OD600: Optical Density (600nM) after 18-24 h growth at 32°C under anaerobic growth conditions ± the standard deviation (SD).
